# Association of neutrophil-to-lymphocyte ratio with clinical, pathological, radiological, laboratory features and disease outcomes of invasive breast cancer patients: A retrospective observational cohort study

**DOI:** 10.1097/MD.0000000000033811

**Published:** 2023-05-17

**Authors:** Sarosh Khan Jadoon, Rufina Soomro, Muhammad Nadeem Ahsan, Raja Muhammad Ijaz Khan, Sadia Iqbal, Farah Yasmin, Hala Najeeb, Nida Saleem, Namiya Cho, Resham, Taha Gul Shaikh, Syeda Fatima Saba Hasan, Muhammad Zain Khalid, Sarosh Alvi, Ahsan Mujtaba Rizvi, Muhammad Sohaib Asghar

**Affiliations:** a Department of General Surgery, CMH Muzaffarabad, Azad Kashmir, Pakistan; b Department of General Surgery, Liaquat National Hospital and Medical College, Karachi, Pakistan; c Department of Nephrology, Dow University of Health Sciences, Karachi, Pakistan; d Department of Internal Medicine, Dow University of Health Sciences, Karachi, Pakistan; e Teaching Assistant, Faculty of Medicine, University of Bakht Al-Ruda, Khartoum, Sudan; f Department of Medicine, Baylor College of Medicine, Houston, TX; g Division of Nephrology and Hypertension, Mayo Clinic-Rochester, Rochester, MN.

**Keywords:** breast, cancer, malignancy, NLR, surgery

## Abstract

Inflammatory conditions play part in the progression of malignancies, and markers signifying growth of these factors can indicate prognosis. Neutrophil-to-lymphocyte (NLR) is used as a marker of subclinical inflammation that may become an integral part of workup to indicate prognosis and associated pathology. This study aims to explore the association of NLR ratio with clinical characteristics, radiological assessment and staging, histopathology, and disease outcomes of breast cancer. A retrospective cohort study was conducted in a tertiary care center to include breast cancer patients that were diagnosed between January 2001 and December 2020. Data including tumor size, lymph nodes, metastasis, histological grading, ER/PR/HER2-neu status, molecular subtypes, clinical staging); nodal findings (sentinel and axillary); pathology from frozen section; and disease outcomes were assessed. Multivariable regression and Kaplan–Meier survival curves were employed to indicate the association of NLR with breast cancer features and disease-free survival. A total of 2050 patients had a median age of 50 years, median NLR levels of 2.14, most common pathology ductal followed by lobular, and most common site of metastasis being lungs followed by bones. Disease-free rate was 7.6%, and a recurrence rate of 1.8%, while 1.6% deaths were reported. NLR was found associated with age, treatment outcomes, tumor size, lymph nodes, metastasis and clinical staging. Other positive correlations were with Ki67 proliferation index, molecular subtypes, and tumor size on frozen section (at transverse and craniocaudal dimensions). Negative correlations were seen with estrogen and progesterone receptors. However, NLR was not found predictable of disease-free survival (*P* = .160). Significant predictors of disease-free survival were histological grading, ER, PR status, molecular subtype, and Ki67 proliferation index. NLR being a readily available marker has shown novel findings in its association with tumor staging, disease outcomes and characteristics of breast malignancy.

## 1. Introduction

Accumulating evidence suggests that chronic inflammation is linked, in general, with tumor progression and is termed as tumor microenvironment where immunocytes and cytokines play a role.^[1]^ Circulating leukocytes such as neutrophils and lymphocytes are recognized as part of the immune reaction to malignancy and promoting tumor growth.^[2,3]^ Heterogeneity in breast cancer is usually caused by factors such as co-morbidities, histopathology, immunochemistry, and molecular subtyping of the tumor. Therefore, certain inflammatory markers such as neutrophil-to-lymphocyte ratio (NLR) because of their chronic inflammatory role are being studied as circulating markers to evaluate prognostic factors as evidence to determine the outcome in patients.^[3]^ Not only with cancers, but high NLR is generally associated with poor survival outcomes in other diseases.^[4,5]^

Numerous studies have found existence of a relationship between systemic inflammatory markers activation and poor prognosis in various types of breast cancer.^[6–8]^ NLR is being used as a marker of inflammation to predict the outcomes due to its potential usefulness, inexpensiveness, and availability. NLR performs better than other leukocyte parameters, due to its stability which is attributable to the fact that it remains unaffected by a variety of physiological and pathological circumstances, and it represents the inflammatory and immune processes simultaneously that co-existed in a diseased patient.^[9]^ Although the exact mechanism behind NLR’s significance in tumor prognosis is unknown, cancer-related chronic inflammation is thought to boost the production of factors that promote carcinogenesis.^[10]^ Though it is not possible to determine whether the malignancy in advanced stages produces more inflammatory mediators, or chronic inflammatory process causes an increase in NLR which accelerates the progression of tumor and metastasis. However, one proposed theory is phagocyte system being part of neutrophils consists of a significant killing mechanism of the pathogens (phagocytosis) with marked potential to release the reactive oxygen species,^[1]^ through which a pro-apoptotic effect takes place. According to the theory, neutrophils proliferate with tumor progression and have various properties that impact tumor cytotoxicity and immune suppression. This explains the mechanism underlying the risk of worse outcomes in malignancy with increased NLR.^[1,9,10]^

Some studies have differentiated benign proliferative breast disease with malignancy based on NLR, as well as it is predictive of febrile neutropenia post-chemotherapy.^[11,12]^ However, the correlation of NLR with various clinicopathological features of breast malignancy is not well understood. Therefore, in this study, we aim to investigate the association of NLR with various prognostic clinicopathological features of breast cancer patients including clinical characteristics, radiological assessment and staging, histopathology, and disease outcomes.

## 2. Materials and Methods

A retrospective cohort study was conducted in a single-center, tertiary care hospital to include histologically confirmed breast cancer patients between January 2001 and December 2020. All patients were at least having a recent follow-up visit between the years 2018 to 2020 except for mortalities. The study was conducted with HIPAA compliance, IRB approval taken from ethical review committee of (Liaquat National Hospital and Medical College), and adhering to the Declaration of Helsinki, with over 2000 patients were recruited and included in the final analysis after non-probability consecutive sampling method. STROBES guidelines were followed in describing the findings.

Female patients with a biopsy-proven diagnosis of breast cancer were included. Inclusion criteria were patients aged between 18 to 90 years with histology confirmed breast cancer of all stages. All patients underwent workup before commencing the treatment either surgical or non-surgical. Patient demographics, tumor characteristics (including tumor size, lymph nodes, metastasis, histological grading, estrogen receptor (ER)/progesterone (PR)/HER2-neu status, molecular subtypes, clinical staging); ultrasound nodal findings (including sentinel and axillary lymph nodes); pathology from frozen section; and disease outcomes were assessed. The American Joint Committee on Cancer classification was used to determine clinical staging of the included patients. The laboratory data including complete blood picture focusing on neutrophil and lymphocytes counts were also obtained. NLR was calculated by dividing absolute neutrophil count by absolute lymphocyte count. Only pretreatment NLR was considered for cross-sectional analysis. The diagnostic kit utilized in our institution for running complete blood picture is CELL-DYN Ruby Hematology Analyzer by Abbott (Abbott Park, IL) which is an automated multi-parameter design utilizing Multi-Angle Polarized Scatter Separation (MAPSS) technology to determine the cell count analysis.

**Figure 1.**
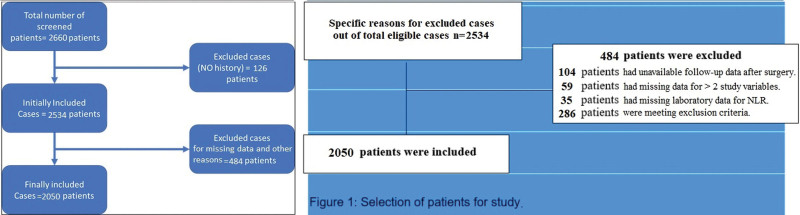
Selection criteria of the patients and number of excluded cases.

Patients with inflammatory breast cancer, ductal carcinoma in situ, and those diagnosed with any systemic or autoimmune or other chronic illness, on steroid therapy, or simultaneously diagnosed with malignancy in any other organ were excluded from the study. Other exclusions were cases with missing clinical data (history/follow-up/>2 study variables/laboratory data). The cases with no more than 2 missing variables were included. Patient selection criteria and total number of excluded cases are shown in Figure 1. Data analysis was performed by SPSS version 25.0 for Windows (Armonk, NY). The qualitative variables such as tumor histopathology and other characteristics were described as percentages and frequency. The quantitative variables such as age and NLR are presented as median and interquartile range (IQR). Correlation with NLR was established using either Mann–Whitney *U* or Kruskal–Willis H test among groups as indicated (Dunn-Bonferroni post hoc method following a significant Kruskal–Wallis test was used). cutoff for NLR was determined by Receiver Operating Characteristics (ROC) as 2.5, and further categorical associations were made through Fisher’s exact test or Chi-Square test depending on applicability. Multivariable regression and Kaplan–Meier survival curves were employed to indicate the association of NLR with breast cancer features and disease-free survival respectively. Significance was obtained by Wald’s methods and log-rank test respectively. A *P* value of less than .05 was considered significant (two-tailed).

## 3. Results

### 3.1. Baseline statistics

A median follow-up period of 20.0 (9.0–44.0) months was observed for a total of 2050 included patients. The maximum follow-up period available was 231 months (i.e., 19.25 years) although very few patients had such long follow-ups which can be a major limitation of this study. Median (IQR) age was 50.0 (41.0–60.0) years with about 55% individuals of less than 50 years. The most likely histopathological diagnosis was invasive ductal carcinoma (72%). Around 6.2% of females were having metastasis at the time of diagnosis (n = 124), with lungs (n = 56) followed by bones (n = 49) being the most likely site for primary metastasis. Disease recurrence was observed in 1.8% (n = 36) patients during follow-up, out of which mostly occurred in contralateral breast (36%) followed by locoregional recurrence (31%). Only 19% (n = 7) of those were systemic recurrences, sites of which were Lungs (n = 2), Brain (n = 2), Bones (n = 2), and Liver (n = 1) respectively as shown in Table 1. Median (IQR) NLR was 2.14 (1.62–2.84), while mean SD was 2.50 (1.89). Hence a cutoff value of 2.50 for NLR was determined by ROC statistics for subgroup analysis of study variables.

**Table 1:** Baseline data of the study population with missing values (n = 2050).

Variables (n = available data/total data)	Characteristics	Descriptive statistics	Missing values (N)
Age (n = 2050)	Median (IQR)	50.0 (41.0–60.0)	0
Age groups	<50 yr	1124 (54.8%)	
	>50 yr	926 (45.2%)	
Histopathology (n = 1706)	Ductal	1232 (72.2%)	344
	Lobular	190 (9.3%)	
	Papillary	43 (2.5%)	
	Mucinous	38 (2.2%)	
	Paget’s disease	18 (1.1%)	
	Metaplastic	12 (0.7%)	
	Phylloides	7 (0.4%)	
	Others	35 (1.7%)	
	Undetermined	131 (7.7%)	
Site of primary metastasis at diagnosis (n = 124)	Lungs	56 (45.1%)	–
	Bones	49 (39.5%)	
	Liver	13 (10.5%)	
	Brain	2 (1.6%)	
	Pelvic	2 (1.6%)	
	Pleura/chest wall	2 (1.6%)	
Disease recurrence (n = 36)	Systemic	7 (19.4%)	–
	Locoregional	11 (30.6%)	
	Local	5 (13.9%)	
	Contralateral	13 (36.1%)	
Site of metastasis on systemic recurrence (n = 7)	Liver	1 (14.3%)	–
	Lungs	2 (28.6%)	
	Brain	2 (28.6%)	
	Bones	2 (28.6%)	
Mode of surgical intervention (n = 2050)	No surgical intervention	344 (16.8%)	0
	Breast-conserving surgery (BCS)	470 (22.9%)	
	Simple mastectomy	480 (23.4%)	
	Modified radical mastectomy (MRM)	756 (36.9%)	
Modality of treatment (n = 2050)	Surgery + adjuvant chemo	625 (30.5%)	0
	Surgery + adjuvant radio	2 (0.1%)	
	Neoadjuvant chemo	45 (2.2%)	
	Surgery alone	72 (3.5%)	
	Chemotherapy alone	19 (0.9%)	
	Radiotherapy + chemotherapy	4 (0.2%)	
	Surgery + chemo + radio	309 (15.1%)	
	Surgery + chemo + radio + hormonal therapy	941 (45.9%)	
	Conservative management/palliative care	33 (1.6%)	
Disease outcome at most recent follow-up (n = 2050)	Ongoing treatment	1826 (89.1%)	0
	Disease free	156 (7.6%)	
	Recurrence	36 (1.8%)	
	Death	32 (1.6%)	
TNM staging (T) (n = 2000)	T0	45 (2.3%)	50
	T1	99 (5.0%)	
	T2	1023 (51.2%)	
	T3	310 (15.5%)	
	T4	523 (26.2%)	
TNM staging (N) (n = 2001)	N0	1016 (50.8%)	49
	N1	851 (42.5%)	
	N2	122 (6.1%)	
	N3	12 (0.6%)	
TNM staging (M) (n = 2000)	M0	124 (6.2%)	50
	M1	1876 (93.8%)	
Clinical staging (n = 1968)	I	82 (4.2%)	82
	II	1068 (54.3%)	
	II A	612 (31.1%)	
	II B	456 (23.2%)	
	III	694 (35.3%)	
	III A	214 (10.9%)	
	III B	470 (23.9%)	
	III C	10 (0.5%)	
	IV	124 (6.3%)	
Histological grading (n = 1146)	1	74 (6.5%)	904
	2	494 (43.1%)	
	3	578 (50.4%)	
Number of sentinel lymph node involved (n = 1706)	0 (none)	1318 (77.3%)	344
	1–2 (limited)	317 (18.6%)	
	≥3 (Extensive)	71 (4.2%	
Number of axillary lymph node involved (n = 1706)	None	1082 (63.4%)	344
	1–3	309 (18.1%)	
	4–10	192 (11.3%)	
	≥11	123 (7.2%)	
Anteroposterior dimension of tumor (on frozen section) (n = 1558)	<2 cm	288 (18.5%)	492
	2–5 cm	989 (63.5%)	
	>5 cm	281 (18.0%)	
Transverse dimension of tumor (on frozen section) (n = 1542)	<2 cm	580 (37.6%)	508
	2–5 cm	865 (56.1%)	
	>5 cm	97 (6.3%)	
Craniocaudal dimension of tumor (on frozen section) (n = 1368)	<2 cm	768 (56.1%)	682
	2–5 cm	573 (41.9%)	
	>5 cm	27 (2.0%)	
Estrogen receptor (ER) (n = 2000)	Negative (0)	570 (28.5%)	50
	Weakly positive (1+)	119 (5.9%)	
	Intermediate (2+)	95 (4.8%)	
	Strongly positive (3+)	1216 (60.8%)	
Progesterone receptor (PR) (n = 2000)	Negative (0)	832 (41.6%)	50
	Weakly positive (1+)	114 (5.7%)	
	Intermediate (2+)	162 (8.1%)	
	Strongly positive (3+)	892 (44.6%)	
HER2/neu receptor (n = 1949)	Negative (0)	975 (50.0%)	101
	Weakly positive (1+)	266 (13.6%)	
	Intermediate (2+)	345 (17.7%)	
	Strongly positive (3+)	363 (18.6%)	
Molecular subtype (n = 1968)	Triple negative	257 (13.8%)	82
	Luminal A	186 (10.0%)	
	Luminal B	1142 (61.1%)	
	HER2/neu positive (only)	283 (15.1%)	
Ki67 proliferation index (n = 1735)	≤14% (low)	470 (27.1%)	315
	15-34% (medium)	494 (28.5%)	
	≥35% (high)	771 (44.4%)	
NLR (n = 2050)	Median (IQR)	2.14 (1.62–2.84)	0

Descriptive data are presented as either median (IQR) or frequency (%).

Where n is either data available for a particular variable, or total; while N is missing data.

BCS = breast-conserving surgery, ER = estrogen receptor, HER2neu = herceptin, MRM = modified radical mastectomy, NLR = neutrophil-to-lymphocyte ratio, PR = progesterone receptor, TNM = tumor size, lymph nodes, metastasis.

### 3.2. Association of NLR with mode of intervention and disease outcomes

**Figure 2.**
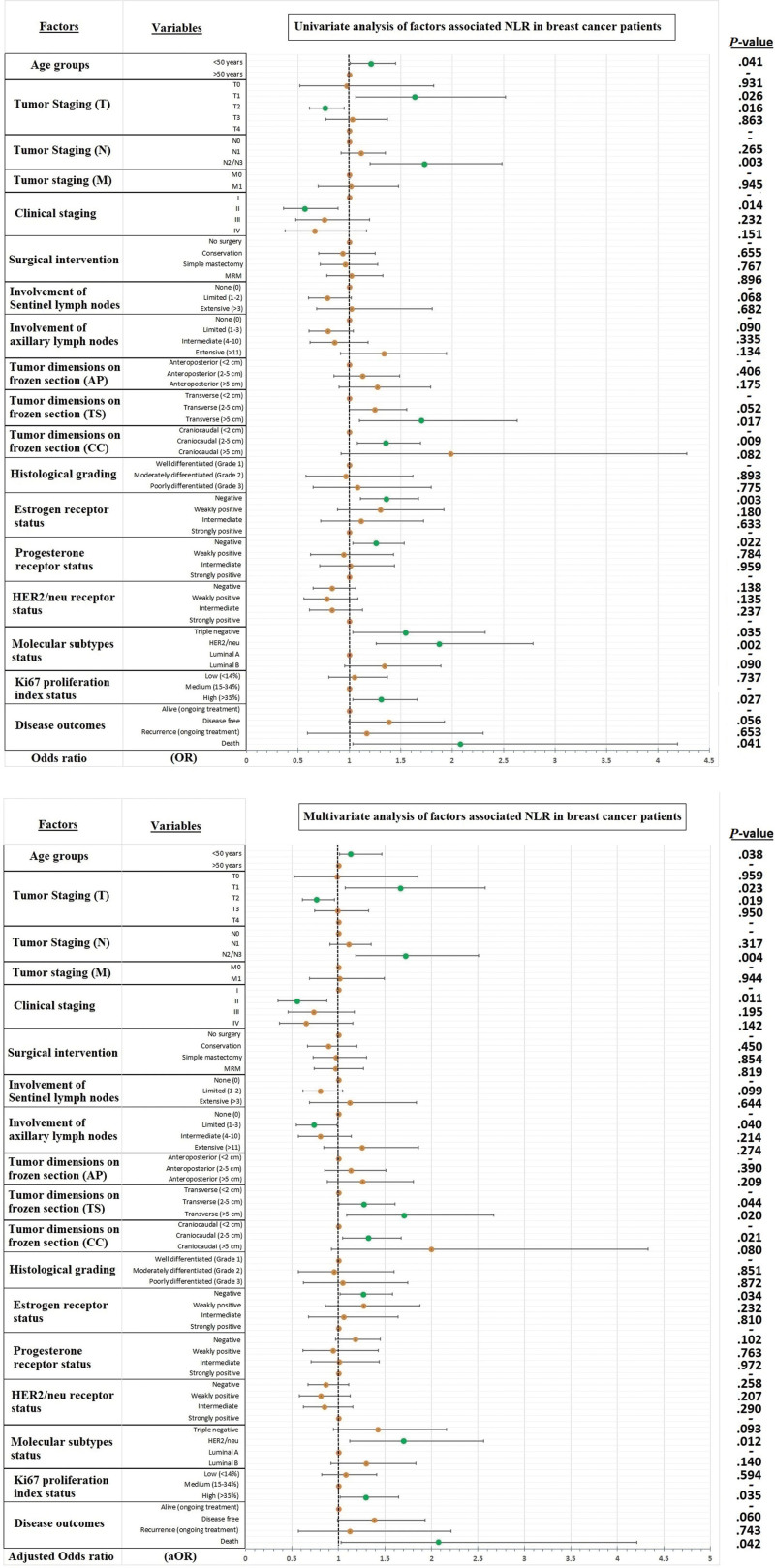
Multivariable regression showing association of NLR with study variables. NLR = neutrophil-to-lymphocyte ratio.

**Figure 3.**
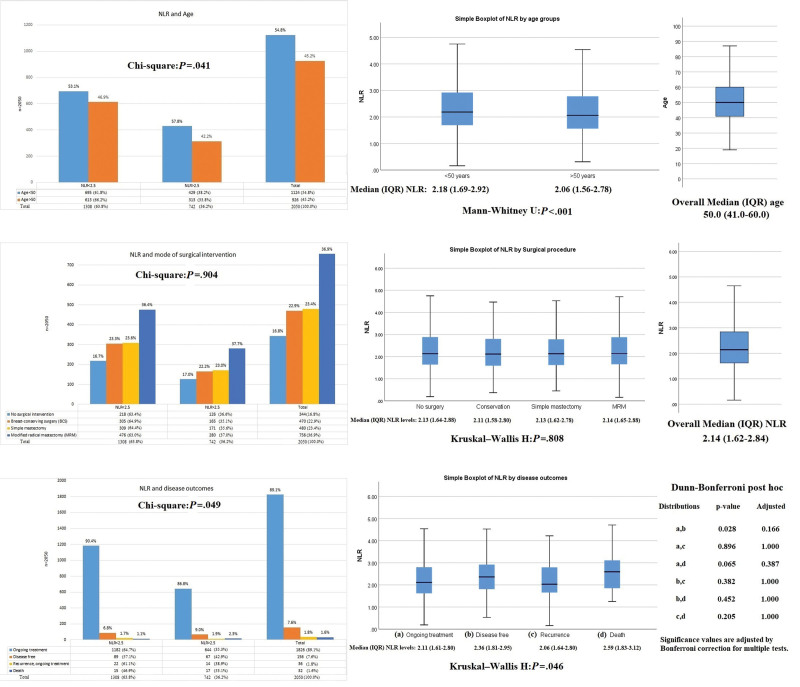
Association of NLR with age, mode of surgical intervention, and disease outcomes. NLR = neutrophil-to-lymphocyte ratio.

Mortality was reported in 1.6% of the study participants (n = 32). A higher NLR is associated with mortality having OR: 2.08 (95% CI: 1.032–4.193, *P* = .041) as shown in Figure 2. Surgery was performed in a total of 1706 individuals (83.2%), rest of them were either on conservative management/palliative care or other modalities (i.e., chemotherapy, radiotherapy, or combination). Most frequently performed surgical modality was modified radical mastectomy (37%), followed by simple mastectomy (23%), and breast conservation surgery (23%) respectively. NLR was not found associated with any particular surgical modality (*P* = .808) as shown in Figure 3.

### 3.3. Association of NLR with staging of malignancy and grade of the tumor

**Figure 4.**
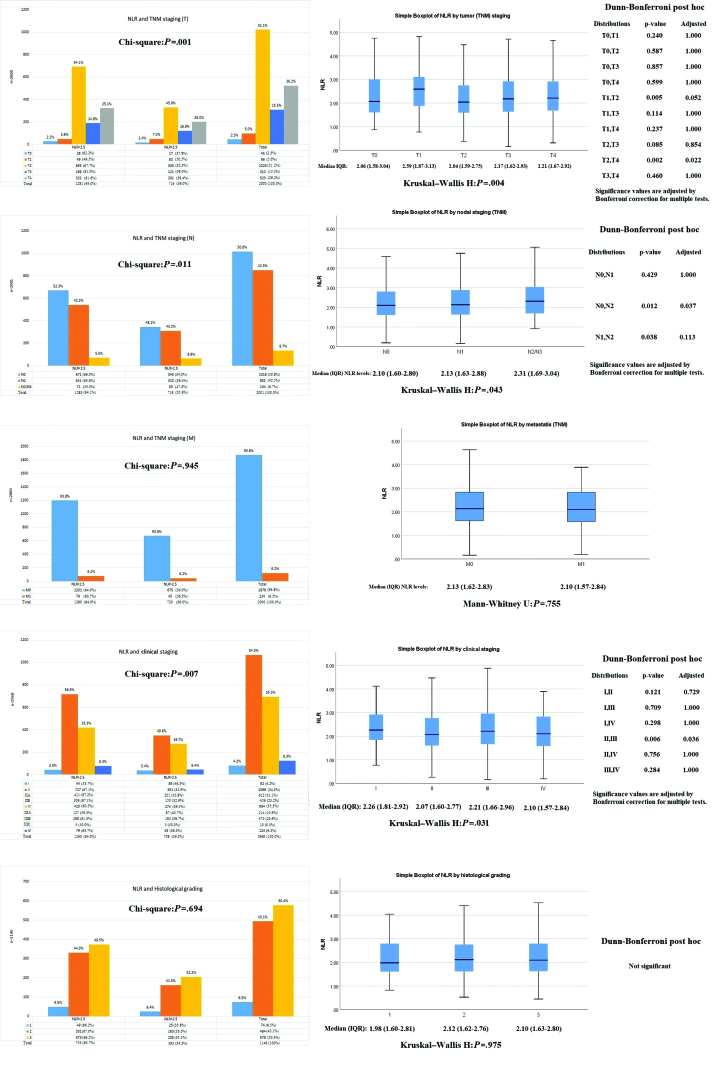
Association of NLR with stage and grade of the patients. NLR = neutrophil-to-lymphocyte ratio.

Among the clinical staging, majority 54% individuals were having stage II disease followed by stage III in 35% (stage II A and III B were highly involved in that order). About 50% had grade 3 disease on histopathological differentiation, while 43% had grade 2 and the rest 7% had grade 1 disease. On tumor size, lymph nodes, metastasis staging, most patients were found with T2 lesions (51%) followed by T4 (26%). On nodal involvement, 42.5% of patients have N1 status, and the remaining 7% had N2/N3. Another 6% had shown metastasis. Elevated NLR was found associated with T1 (OR: 1.63 [1.06–2.52], *P* = .026) and N2/N3 (OR: 1.73 [1.20–2.48], *P* = .003), while in clinical stage II NLR was found decreased (*P* = .031). No such difference can be found in histological grading (*P* = .694) and metastatic disease (*P* = .945) as shown in Figure 4.

### 3.4. Association of NLR with sentinel/axillary lymph node status and tumor dimensions on frozen section

**Figure 5.**
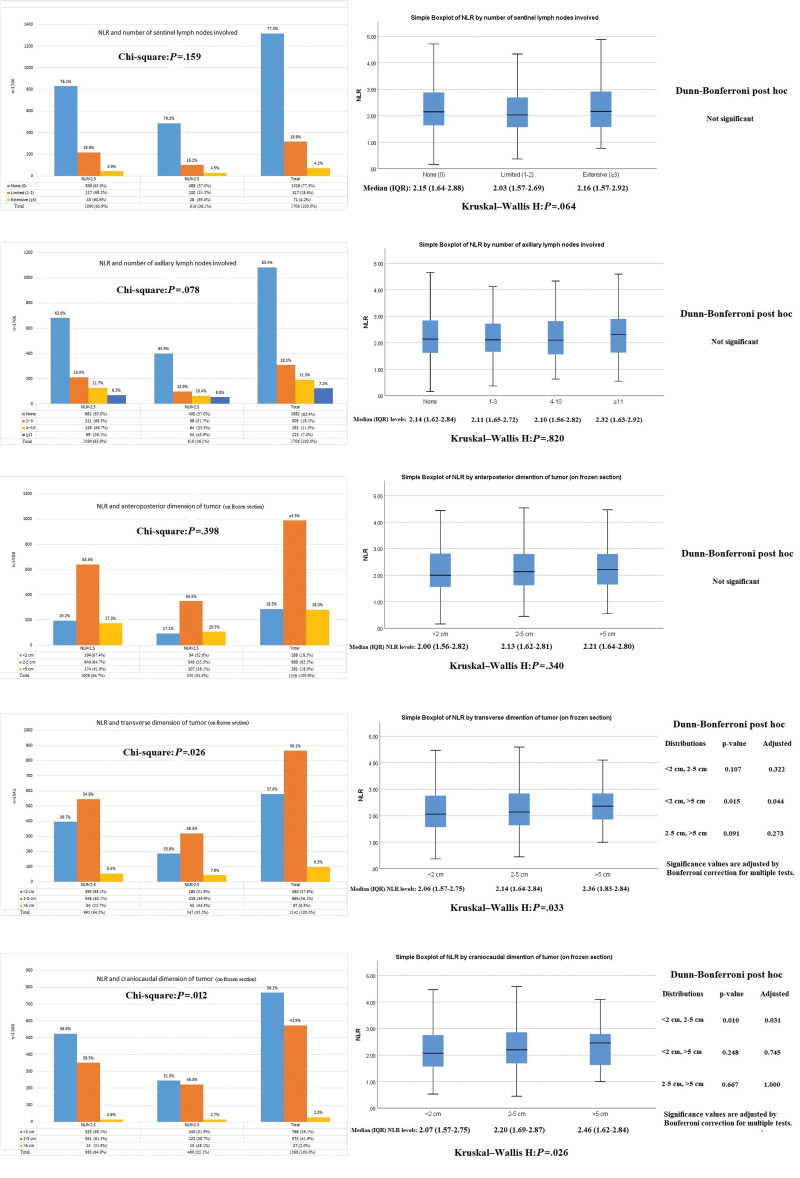
Association of NLR with lymph node status and tumor dimensions on frozen section. NLR = neutrophil-to-lymphocyte ratio.

With respect to sentinel lymph node involvement, more than three-fourths of the patients had nil while rest 19% and 4% had limited and extensive involvement respectively. Axillary status was also clear in 63% of those patients, while another 18% had 1 to 3 axillary nodes positive, 11% had 4 to 10 nodes positive, and rest 7% had > 11 nodes. NLR was not found associated with either sentinel or axillary node status. However, at tumor dimensions on frozen section, elevated NLR was significantly correlating with increasing transverse and craniocaudal dimensions of tumor mass. Further, this correlation was not significant at anteroposterior dimension as shown in Figure 5.

### 3.5. Association of NLR with molecular, hormonal and proliferation index subsets

**Figure 6.**
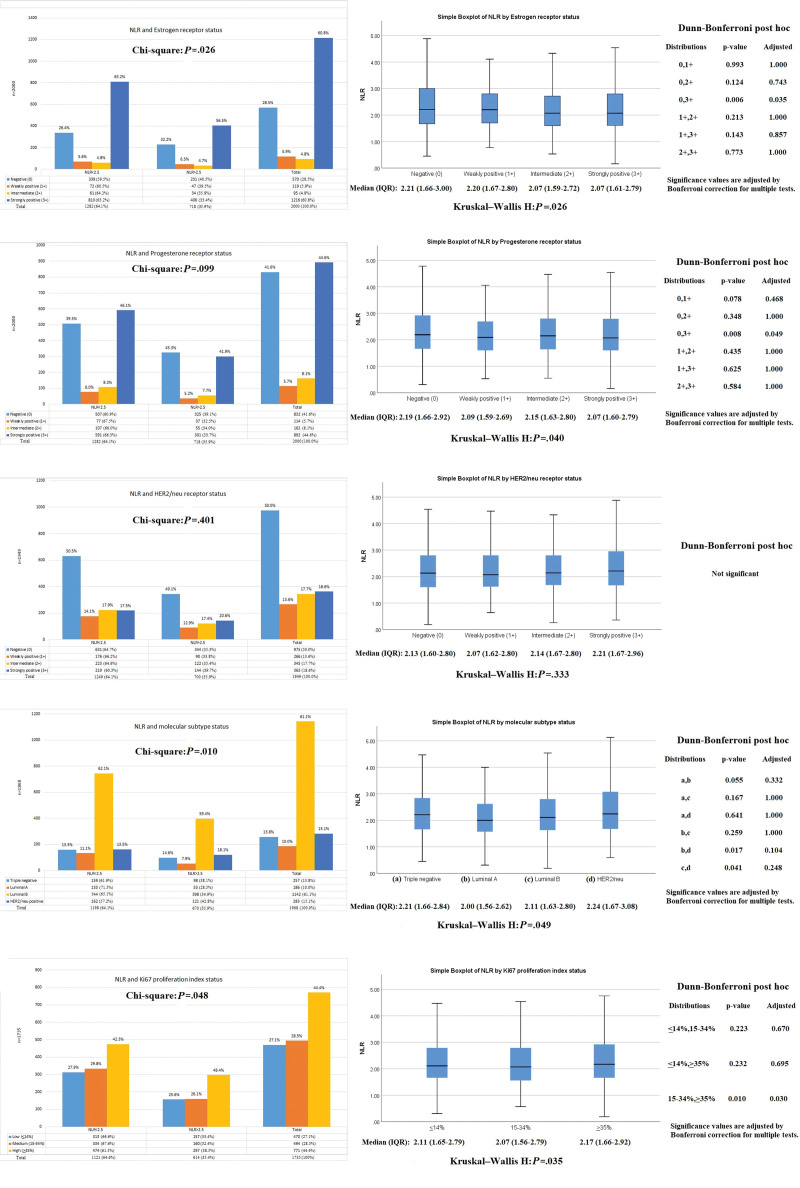
Association of NLR with molecular, hormonal and Ki67 proliferation index. NLR = neutrophil-to-lymphocyte ratio.

ER was positive in around 71.5% of patients, among whom 5.9% showed weak positivity, 4.8% showed intermediate while 60.8% have shown strongly positive expression on tumor cells. Similarly, PR was positive in 58.4% of the participants, among which 5.7% were weakly positive, 8.1% were intermediate, and rest 44.6% were strongly positive. HER2/neu was also positive in one-half of those among which data was available, and equally distributed into weak/strongly positive. Among the molecular subtypes, Luminal B tumors were most frequently found (61%) followed by HER2neu positive exclusively (15.1%) and triple negative cancers (13.8%). Luminal subtype was found in only 10% of individuals. Ki67 proliferation index showed high activity in 44%, medium in 28%, and low in 27% of the patients. Interestingly, NLR showed a negative correlation with ER and PR positivity with higher NLR tends to be associated with a negative ER (*P* = .026) and PR expression (*P* = .040) respectively. Similarly, slightly higher median NLR was also observed in those with triple negative and HER2/neu positive cancers (*P* = .049). High Ki67 index was also associated with elevated NLR (*P* = .035) as shown in Figure 6. The adjusted odds of NLR for HER2neu receptor positivity and high Ki67 index were 1.69 [1.12–2.56], (*P* = .012) and 1.29 [1.02–1.64], (*P* = .035) respectively.

### 3.6. Disease-free predictors on survival analysis

**Figure 7.**
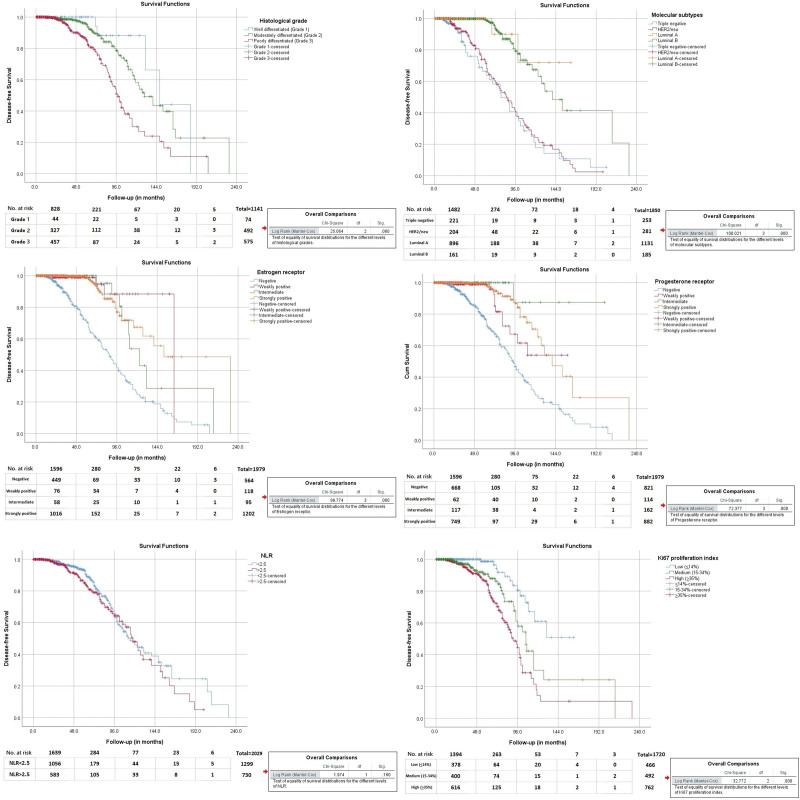
Kaplan–Meier survival analysis to indicate disease-free survival of study variables.

Despite NLR being markedly increased in those with mortality, it was not found predictable of disease-free survival on Kaplan–Meier survival analysis (*P* = .160). Significant predictors of disease-free survival were histological grading (*P* < .001), ER (*P* < .001), PR status (*P* < .001), molecular subtype being either triple negative or HER2/neu positive (*P* < .001), and higher Ki67 proliferation index (*P* < .001) as shown in Figure 7. In all these clinical, pathological and laboratory correlations with NLR, it is found that NLR was not associated with certain good prognostic markers while showing elevated levels in some of the bad prognostic indicators.

## 4. Discussion

Many researchers previously have seen the association of NLR with adverse prognostic outcomes in breast malignancy. A meta-analysis of over 8 studies reflected its utility as a predictor of overall survival with a hazard ratio (HR) of 2.28 (1.08–4.80), especially in Caucasian population.^[13]^ Disease-free survival was also associated with elevated NLR with an HR of 1.38 (1.09–1.74). This was contrary to our findings as we did not reach statistical significance for disease-free survival. A study conducted on 608 Chinese breast cancer patients had a lower median NLR than our study (1.75) however shown a significant association with overall survival rate at a cutoff value 2.56 (*P* < .001). Disease-free survival was not found significant (*P* = .084), similar to our study.^[14]^ Relapse free survival was also associated with NLR reported by Chen et al^[15]^ with HR: 1.57 (1.05–3.57) and Fujimoto et al^[16]^ with HR: 3.52 (1.61–7.32). Similarly, recurrence free survival was also reported to be associated with NLR by Koh et al^[17]^ and Chae et al^[18]^

Disease-free survival was also positively associated with elevated NLR in studies from Japan,^[19,20]^ China,^[21–27]^ Turkey,^[28,29]^ Austria,^[30]^ Belgium,^[31]^ Korea,^[32–34]^ Italy,^[35,36]^ Spain,^[37,38]^ and Costa Rica;^[39]^ whereas studies from Cihan et al (*P* = .41),^[40]^ Ulas et al (*P* = .45,^[41]^ Suppan et al (*P* = .363),^[42]^ Qian et al (*P* = .535),^[43]^ Takeuchi et al (*P* = .11),^[44]^ and Patel et al (*P* = .77),^[45]^ did not reach statistical significance for pretreatment NLR and disease-free survival. Decreased overall survival and higher mortality was also predicted by elevated NLR by various studies.^[16,17,19,20,27,29,38,46–50]^ Breast cancer-specific survival was also predicted by elevated NLR by a few studies.^[15,19,51]^ Higher risk of relapse with elevated NLR was also reported by one study from Belgium.^[52]^ Metastasis free survival was reported by Orditura et al^[53]^ in which lower NLR denotes higher metastasis free survival rates. Similarly, Kim et al^[54]^ also observed metastasis free survival affected by elevated NLR at HR: 1.92 (1.09–3.37), whereas Ferroni et al^[36]^ observed that NLR had most prognostic value in stage 1 disease where it significantly stratified breast cancer patients with development of distant metastasis.

On the contrary, Rimando et al^[55]^ found no associations between elevated NLR and all-cause or breast cancer-specific mortality. However, among patients without metastasis, NLR was independently associated with all-cause mortality, with HR: 2.31 (1.10–4.86) but not with breast cancer-specific mortality (in non-metastatic breast cancer patients).^[55]^ With respect to overall survival following recurrence, Iwase and his colleague compared pretreatment NLR levels with those at the time of recurrence and concluded that high NLR demonstrated poor survival upon recurrence especially in triple negative breast cancers.^[56]^ These findings were similar to those reported by Lee et al^[34]^ in terms of overall and disease-free survival in the Korean subset of triple negative breast cancers. Similar results were predicted by Chinese researchers in studying non-metastatic triple negative breast cancer,^[23,26]^ and in non-metastatic HER2/neu positive cancers.^[25]^ In Poland, overall predicted survival was found borderline significant with elevated NLR (*P* = .053), but it was significant in triple negative subset of their cohort (*P* = .034).^[57]^ Yao et al^[14]^ reported poor survival with elevated NLR in both triple negative and Luminal A subset of Chinese breast cancer population, while Liu et al reported the same in triple negative and HER2/neu positive cancers.^[21]^ No NLR prediction of survival with triple negative breast cancers was concluded in a multicentric study from USA.^[45]^ In Korean population with ER/PR positive and Her2/neu negative cancers, NLR remained independently predictive of survival.^[17]^ Data from Canada also suggested prognostic value of NLR in patients with triple negative breast cancer,^[49]^ while another study predicted it in both triple negative and HER2/neu positive cases.^[50]^

Authors from Japan gave an interesting narrative to this association, by linking it to high absolute lymphocytes counts only, while those patients with low absolute lymphocyte counts were showing no significance of NLR in predicting survival.^[16]^ Previously, this narrative was countered by Azab et al^[47]^ from USA in their retrospective analysis of 437 patients, where they found out that elevated NLR was equally significant in predicting mortality with or without lymphopenia. Now coming to the clinicopathological correlation of breast malignancy features, various studies conducted have linked them with the NLR. In our study, there was a significant variation in NLR association with various disease characteristics. We found a strong link with disease staging, similar to how Elyasinia et al^[58]^ found a higher NLR ratio with a higher clinical stage, but in contrast, they found no significant relationship with ER, PR, or HER2/neu.^[29]^ Our results also negate the study of Yilmaz et al^[59]^ who claimed no significance between preoperative NLR with breast cancer subtypes. But we found NLR higher in triple negative, and HER2/neu positive cases than the luminal A and B subtypes. Our findings are consistent with multiple studies in showing a significant relationship between NLR and the T stage of the disease or tumor size.^[15,16,26,28,44,50,51]^ NLR was also found associated with age,^[19,23,26,33,51,57]^ lymph node status,^[26,28]^ distant metastasis,^[28,33,50]^ clinical staging,^[15,19,26,28,33]^ histopathological grading,^[19,26,50]^ PR status,^[50]^ Ki67 proliferation index,^[16]^ and HER2/neu status.^[49,51]^

Poor survival was found associated with age,^[23,32,54]^ lymph node status,^[15,16,19,23,24,26,28,32,33,41,42,44,51,54,57]^ tumor size,^[16,24,26,28,29,31–33,42,44,54,57]^ clinical staging,^[24,28,34,41,49,55]^ histopathological grading,^[15,24,26,28,33,41,44,55]^ distant metastasis,^[33]^ HER2/neu positivity,^[28,40,54]^ ER negative,^[16,31,51,57]^ PR negative,^[24,31,33,42]^ High Ki67 index,^[16]^ and triple negative cancers.^[28,32,55,56]^ Some studies do not show any influence of chemotherapy or mode of treatment other than surgery with varying NLR.^[16,26,48,59]^ For instance, patients on radiotherapy showed significantly varying NLR in one study.^[46]^ With respect to surgical intervention, Koh et al^[50]^ and Huszno et al^[57]^ reported high NLR with no surgery patients, in opposed to surgical patients while the current study found no difference in mode of surgical intervention. Qiu et al^[26]^ concluded low NLR in breast conservation surgery patients in comparison to radical mastectomy. The novel finding of our study was negative correlation of NLR with ER and PR positivity. Previously many studies showed no such association,^[15,19,28,51]^ except for Geng et al^[27]^ who found higher NLR with hormone positive cancer, opposing our findings. Huszno et al^[57]^ significantly reported high NLR with ER receptor positivity opposing our findings. Borderline significance was also reported by Cho et al^[33]^ in high NLR with PR receptor positivity. Molecular subtype was also not found associated with NLR in Austrian and Turkish studies.^[42,60]^ Lastly, Ki67 proliferation index was not found associated with NLR in multiple studies and incidentally their cohorts were triple negative breast cancers,^[20,29,34]^ but we have found positive correlation among the 2 i.e., high Ki67 with higher NLR. Hence, inference can be made that proliferative index correlates with NLR in hormone receptor positive breast cancers, which constitutes most of our cohort. This correlation signifies the importance of tumor immune microenvironment that constitute proliferation of abnormal cells and its enhanced activity is marked by noninvasive blood cell markers elevation including NLR.

The limitations of the current study include a single-center design and that the data regarding histopathology is not available for every patient in this analysis. Another limitation is the inability to access the specific chemotherapy from the oncology department, the details of which could have guided the management regime of these patients. Therefore, further studies to identify the role of NLR in prognostic outcomes and different chemotherapy regimens are still warranted. NLR can also be influenced by race,^[40,45]^ modes of chemotherapy,^[40,47]^ and other factors hence affecting the generalizability of the findings. The majority of cases included were still going through treatment hence disease-free rate might be under-reported in our analysis leading to inadequate survival proportions. The median follow-up period for this study is 20.0 months. However, the patient enrolled in the study from 2001 to 2020. The follow-up period is too short compared to the enrolled period. Hence, it is difficult to analyze and conclude recurrence and survival results with such a short follow-up period (i.e., median f/u 20 months) as well as distinguish between overall survival and breast cancer specific survival. All patients were needed to have at least 1 recent follow-up visit between the years 2018-2020 which makes the inclusion criteria tight enough for screening cases. Based on these strict screening, we noticed that our cases were mostly recent diagnosed, and/or started therapy, with limited follow-up time. Further, less recurrence events occurred which would result in unreliable results, given the good prognosis of breast cancer. Some missing data of clinical and pathological features were also adjusted, and this indicates the study could suffer from different degrees of bias. Another drawback is the lack of stratification with respect to already established prognostic variables in the Kaplan–Meier survival analyses. For example we do not have information whether the significance within the luminal B subtype is independent of tumor stage and use of systemic treatment.

## 5. Conclusions

Inflammatory conditions play part in the progression of malignancies, and markers signifying growth of these factors can indicate prognosis. NLR is used as a marker of subclinical inflammation that can significantly correlate with certain features of breast malignancy and may become an integral part of workup to indicate prognosis and associated pathology. NLR can be easily assessed with the disease characteristics and may indicate poor outcomes in breast malignancy. Therefore, pretreatment NLR being readily available, could be used as a marker and has shown novel findings in its association with tumor staging, disease outcomes, and characteristics of breast malignancy. For further studies regarding NLR, it is crucial to determine what the mechanism is underneath its relation with breast cancer treatment. Unveiling such mechanisms will provide possible improvements in breast cancer treatment and outcomes. It would be more interesting to see some translational data on the microenvironment in a more specific cohort with known treatment details. However, this study did not draw positive results between NLR and survival outcomes, and NLR has no prognostic significance with relation to disease-free survival, so the implications of this study for clinical practice is uncertain as the prognostic and diagnostic value of NLR seems undermined.

## Author contributions

**Conceptualization:** Sarosh Khan Jadoon, Rufina Soomro.

**Data curation:** Sarosh Khan Jadoon, Nida Saleem.

**Formal analysis:** Muhammad Nadeem Ahsan, Muhammad Sohaib Asghar.

**Investigation:** Sadia Iqbal.

**Methodology:** Resham, Taha Gul Shaikh.

**Project administration:** Rufina Soomro, Sarosh Alvi.

**Resources:** Hala Najeeb, Syeda Fatima Saba Hasan.

**Software:** Muhammad Nadeem Ahsan, Raja Muhammad Ijaz Khan.

**Supervision:** Rufina Soomro, Ahsan Mujtaba Rizvi.

**Validation:** Raja Muhammad Ijaz Khan.

**Visualization:** Farah Yasmin, Taha Gul Shaikh.

**Writing – original draft:** Syeda Fatima Saba Hasan, Muhammad Sohaib Asghar.

**Writing – review & editing:** Namiya Cho, Muhammad Zain Khalid, Ahsan Mujtaba Rizvi.
